# Supplementary material for: Opn5L1 is a retinal receptor that behaves as a reverse and self-regenerating photoreceptor
Source: Nat Commun. 2018 Mar 28;9:1255. doi: 10.1038/s41467-018-03603-3 (PMC5871776; doi:10.1038/s41467-018-03603-3)
Supplement: Supplementary file 1 — Supplementary Information [file 41467_2018_3603_MOESM1_ESM.pdf]

Supplementary Information for:

Opn5L1 is a retinal receptor that behaves as a reverse and self-regenerating photoreceptor

Authors: Keita Sato[a], Takahiro Yamashita[b], Hideyo Ohuchi[a], Atsuko Takeuchi[c], Hitoshi Gotoh[d], Katsuhiko Ono[d], Misao Mizuno[e], Yasuhisa Mizutani[e], Sayuri Tomonari[f], Kazumi Sakai[b], Yasushi Imamoto[b], Akimori Wada[g] and Yoshinori Shichida[b,h,1]

Affiliations: [a]Department of Cytology and Histology, Okayama University Graduate School of Medicine, Dentistry and Pharmaceutical Sciences, Okayama 700-8558, Japan; [b]Department of Biophysics, Graduate School of Science, Kyoto University, Kyoto 606-8502, Japan; [c]Division of Analytical Laboratory, Kobe Pharmaceutical University, Kobe 658-8558, Japan; [d]Department of Biology, Kyoto Prefectural University of Medicine, Kyoto 603-8334, Japan; [e]Department of Chemistry, Graduate School of Science, Osaka University, Osaka 560-0043, Japan; [f]Division of Chemical and Physical Analyses, Center for Technical Support, Institute of Technology and Science, Tokushima University, Tokushima 770-8506, Japan; [g]Department of Organic Chemistry for Life Science, Kobe Pharmaceutical University, Kobe 658-8558, Japan; [h]Research Organization for Science and technology, Ritsumeikan University, Kusatsu, Shiga 525-8577, Japan.

[1] To whom correspondence should be addressed:

shichida@rh.biophys.kyoto-u.ac.jp

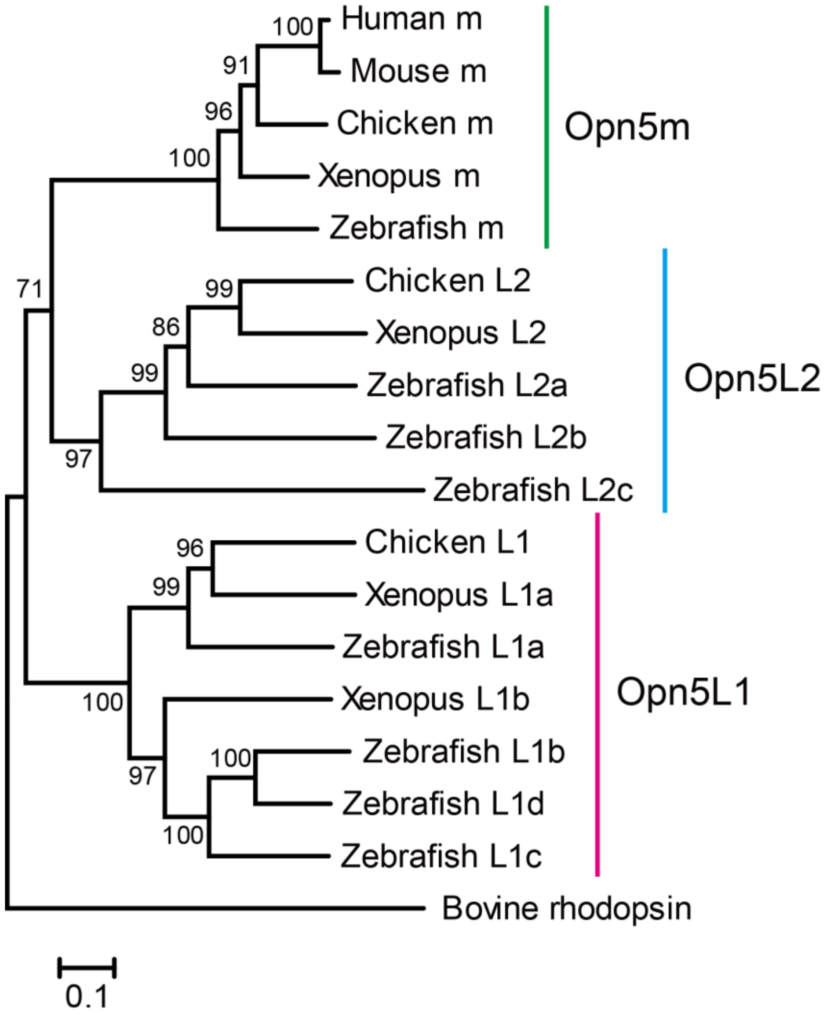

Supplementary Figure 1 Phylogenetic tree of Opn5 proteins

Amino acid sequences of Opn5 proteins found in human, mouse, chicken, Xenopus, and zebrafish were aligned by MAFFT[1], and the phylogenetic tree was constructed by the neighbor-joining method using MEGA6 software[2]. NCBI accession numbers are as follows: human Opn5m, NP_859528; mouse Opn5m, NP_861418; chicken Opn5m, NP_001124215; Xenopus Opn5m, XP_002936036; zebrafish Opn5m, NP_001186975; chicken Opn5L2, NP_001156364; Xenopus Opn5L2, XP_002933633; zebrafish Opn5L2a, XP_001341992; zebrafish Opn5L2b, XP_698118; zebrafish Opn5L2c,

XP_009292879; chicken Opn5L1, XP_419178; Xenopus Opn5L1a, XP_002934176; Xenopus Opn5L1b, NP_001072846; zebrafish Opn5L1a, XP_698966; zebrafish Opn5L1b, NP_001038457; zebrafish Opn5L1c, XP_002666449; zebrafish Opn5L1d, XP_001340602.

a

```
                          ....|....| ....|....| ....|....| ....|....| ....|....|
                                  10         20         30         40         50
Chicken Opn5L1            MD-------- ----PSF--G NSTFQSKITE AADIVVGTCY MVFGICSLCG
Opn5L1N                   MAGNSSYREE SGYIPHYERD SDPFASKLSR EADIVVGTCY MVFGICSLCG
Opn5L1NC                  MAGNSSYREE SGYIPHYERD SDPFASKLSR EADIVVGTCY MVFGICSLCG
Opn5L1_XaFLAGicl3         MAGNSSYREE SGYIPHYERD SDPFASKLSR EADIVVGTCY MVFGICSLCG
X. tropicalis Opn5m       MAGNSSYREE SGYIPHYERD SDPFASKLSR EADIFAGVYL MAIGILSTLG
                          ....|....| ....|....| ....|....| ....|....| ....|....|
                                  60         70         80         90        100
Chicken Opn5L1            NSILLYISYK KKHLLKPAEY FIINLAISDL AMTLTLYPLA VTSSLSHRWL
Opn5L1N                   NSILLYISYK KKHLLKPAEY FIINLAISDL AMTLTLYPLA VTSSLSHRWL
Opn5L1NC                  NSILLYISYK KKHLLKPAEY FIINLAISDL AMTLTLYPLA VTSSLSHRWL
Opn5L1_XaFLAGicl3         NSILLYISYK KKHLLKPAEY FIINLAISDL AMTLTLYPLA VTSSLSHRWL
X. tropicalis Opn5m       NGYVIYMACS RKKKLRPAEI MTINLAVCDL GISVTGKPFA IVSCFSHRWV
                          ....|....| ....|....| ....|....| ....|....| ....|....|
                                 110        120        130        140        150
Chicken Opn5L1            YGKHICLFYA FCGLFFGICS LSTLTLLSVV CCLKICFPAY GNRFRRKHGQ
Opn5L1N                   YGKHICLFYA FCGLFFGICS LSTLTLLSVV CCLKICFPAY GNRFRRKHGQ
Opn5L1NC                  YGKHICLFYA FCGLFFGICS LSTLTLLSVV CCLKICFPAY GNRFRRKHGQ
Opn5L1_XaFLAGicl3         YGKHICLFYA FCGLFFGICS LSTLTLLSVV CCLKICFPAY GNRFRRKHGQ
X. tropicalis Opn5m       FGWNACRWYG WAGFFFGCGS LITLTVVSLD RYLKICHLRY GTWLKRRHAF
                          ....|....| ....|....| ....|....| ....|....| ....|....|
                                 160        170        180        190        200
Chicken Opn5L1            ILIACAWTYA AIFACSPLAH WGEYGEEPYG TACCIDWQST NVDVMSMSYT
Opn5L1N                   ILIACAWTYA AIFACSPLAH WGEYGEEPYG TACCIDWQST NVDVMSMSYT
Opn5L1NC                  ILIACAWTYA AIFACSPLAH WGEYGEEPYG TACCIDWQST NVDVMSMSYT
Opn5L1_XaFLAGicl3         ILIACAWTYA AIFACSPLAH WGEYGEEPYG TACCIDWQST NVDVMSMSYT
X. tropicalis Opn5m       IALAVIWAYA TLWATLPLVG VGNYAPEPFG TTCTLDWWLA QASVKGQIFV
                          ....|....| ....|....| ....|....| ....|....| ....|....|
                                 210        220        230        240        250
Chicken Opn5L1            VVLFVLCFIL PCGVIVTSYS LILVTVKESR KAVEQ----- -------HVS
Opn5L1N                   VVLFVLCFIL PCGVIVTSYS LILVTVKESR KAVEQ----- -------HVS
Opn5L1NC                  VVLFVLCFIL PCGVIVTSYS LILVTVKESR KAVEQ----- -------HVS
Opn5L1_XaFLAGicl3         VVLFVLCFIL PCGVIVTSYS LILVTVKESR KAVEQIEGRD YKDDDDKHVS
X. tropicalis Opn5m       LSMLFFCLLF PTMVIVFSYA KIIAKVKSSA KEVAH----- -------FDT
                          ....|....| ....|....| ....|....| ....|....| ....|....|
                                 260        270        280        290        300
Chicken Opn5L1            GPTRINNVQT ITAKLSIAVC IGFFAAWSPY AIIAMWAAFG SIDKIPPLAF
Opn5L1N                   GPTRINNVQT ITAKLSIAVC IGFFAAWSPY AIIAMWAAFG SIDKIPPLAF
Opn5L1NC                  GPTRINNVQT ITAKLSIAVC IGFFAAWSPY AIIAMWAAFG SIDKIPPLAF
Opn5L1_XaFLAGicl3         GPTRINNVQT ITAKLSIAVC IGFFAAWSPY AIIAMWAAFG SIDKIPPLAF
X. tropicalis Opn5m       RNQNNHTLEI KLTKVAMLIC AGFLIAWFPY AVVSVWSAFG QPDSIPIELS
                          ....|....| ....|....| ....|....| ....|....| ....|....|
                                 310        320        330        340        350
Chicken Opn5L1            AIPAVFAKSS TLYNPIIHLL L--KPNFRSN IAKDFTVIQQ LCVRCCFCVK
Opn5L1N                   AIPAVFAKSS TLYNPIIHLL L--KPNFRSN IAKDFTVIQQ LCVRCCFCVK
Opn5L1NC                  AIPAVFAKSS TLYNPIIHLL LDCKP----- ---------- ---ACCKKDK
Opn5L1_XaFLAGicl3         AIPAVFAKSS TLYNPIIHLL LDCKP----- ---------- ---ACCKKDK
X. tropicalis Opn5m       VVPTMMAKSA SMYNPIIYQV IDCKP----- ---------- ---ACCKKDK
                          ....|....| ....|....| ....|....| ....|....| ....|....|
                                 360        370        380        390        400
Chicken Opn5L1            ELQTYRSTFN TGLRTFKGKN ESSCNALPIM EGCSYFPSEK GSHTFECFKS
Opn5L1N                   ELQTYRSTFN TGLRTFKGKN ESSCNALPIM EGCSYFPSEK GSHTFECFKS
Opn5L1NC                  SLKETSQVAP A--------- ---------- ---------- ----------
Opn5L1_XaFLAGicl3         SLKETSQVAP A--------- ---------- ---------- ----------
X. tropicalis Opn5m       SLQNTTSRVY T-ISTFRKST TSAR------ ---------- ----------
                          ....|....| ....|....| ....|....| ....|....| ....|....|
                                 410        420        430        440        450
Chicken Opn5L1            YPNCFQERLS TMGCHLQDCE SLENDLQVEV TQGSRNSMKV VEQEEKSTEL
Opn5L1N                   YPNCFQERLS TMGCHLQDCE SLENDLQVEV TQGSRNSMKV VEQEEKSTEL
Opn5L1NC                  ---------- ---------- ---------- ---------- ----------
Opn5L1_XaFLAGicl3         ---------- ---------- ---------- ---------- ----------
X. tropicalis Opn5m       ---------- ---------- ---------- ---------- ----------
                          ....|....| ....|....| ....|..
                                 460        470
Chicken Opn5L1            DNLEITLEAV PVSCTFTDL- -------
Opn5L1N                   DNLEITLEAV PVSCTFTDLE TSQVAPA
Opn5L1NC                  ---------- ---------- -------
Opn5L1_XaFLAGicl3         ---------- ---------- -------
X. tropicalis Opn5m       ---------- ---------- -------
```

b

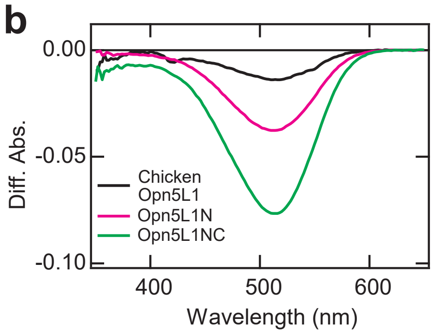

c

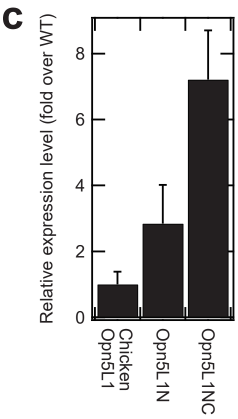

Supplementary Figure 2 Improvement of expression yield of chicken Opn5L1 by replacement of its N- and C-termini with those of *X. tropicalis* Opn5m

(a) Alignment of amino acid sequences of Opn5 proteins. Blue letters show the N- and C-terminal regions of chicken Opn5L1 that were replaced with the corresponding regions of *X. tropicalis* Opn5m to improve its expression yield. Green letters indicate the epitope sequence of anti-rhodopsin Rho1D4 antibody. Magenta letters indicate the factor Xa recognition site and FLAG tag sequence inserted in intracellular loop 3, which are used for cleavage of the protein and detection of the FLAG-tagged protein bands in western blotting analysis (see Supplementary Fig. 10). (b) Difference spectra calculated from the spectra recorded before and after light irradiation of the crude extracts of chicken Opn5L1 proteins. Opn5L1N: chicken Opn5L1 whose N-terminal sequence was replaced by that of *X. tropicalis* Opn5m, Opn5L1NC: chicken Opn5L1 whose N- and C- terminal sequences were replaced by the corresponding regions of *X. tropicalis* Opn5m. These replacements did not affect the absorption maximum of the spectrum, and effectively increased the expression yield of chicken Opn5L1 (see also Supplementary Fig. 3). (c) Expression levels of Opn5L1 variants. In the present study, Opn5L1NC was used in all of the experiments, except for the G protein activation assay, where only N-terminus-replaced Opn5L1 (Opn5L1N) was used (Fig. 1c, j and Supplementary Fig. 4).

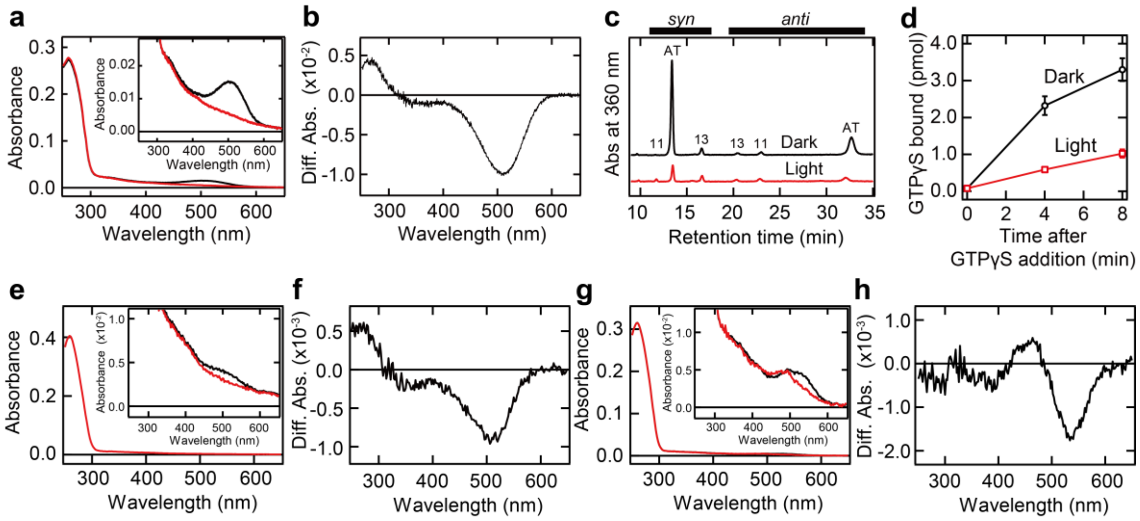

Supplementary Figure 3 Molecular characteristics of Opn5L1 bearing native N- and C-termini

(a) Absorption spectra of the full-length Opn5L1 containing native N- and C-termini. The Opn5L1 sample was purified after incubation with all-*trans*-retinal. Absorption spectra were recorded before (black) and after (red) irradiation with >500 nm light for 2 min. The spectra were recorded at 0 °C. (inset) Enlarged view of the spectra in the visible spectral region. (b) Difference spectrum calculated by subtracting the spectrum recorded after the irradiation from that recorded before the irradiation. (c) HPLC patterns of the retinaloximes extracted from the Opn5L1 sample before (black) and after (red) irradiation with >500 nm light for 2 min. (d) G protein activities of the Opn5L1 before (black) and after (red) >500 nm light irradiation for 2 min. The activities were assayed at 20 °C. Data points represent mean values ± s.d. (n=3). (e-h) Changes of spectral properties by C188T mutation in the full-length Opn5L1 containing native N- and C- termini. Absorption spectra of L118F (e) and L118F/C188T (g) mutants were recorded before (black) and after (red) irradiation with >500 nm light for 2min. The spectra were recorded at 0 °C. (inset) Enlarged view of the spectra in visible

spectral region. Difference spectra of L118F (f) and L118F/C188T (h) mutants were calculated by subtracting the spectrum recorded after the irradiation from that recorded before the irradiation. We constructed a C188T mutant having native N- and C-termini. However, we were unable to investigate the properties of the mutant because of its low expression yield. Therefore, we tried to express the C188T mutant by adding another mutation and finally found that L118F/C188T mutant was reasonably well expressed. L118F mutant showed spectral changes identical to those of wild-type (f). Absorbance at about 480 nm was increased by C188T mutation in the difference spectrum (h), indicating that C188 is necessary for the formation of the 270 nm-absorbing product even in the Opn5L1 having native N- and C- termini.

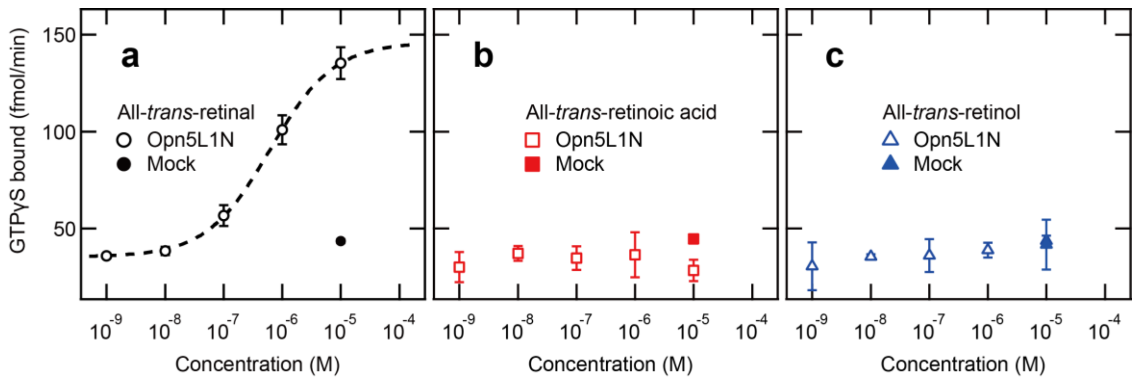

Supplementary Figure 4 All-trans-retinal is an agonist of Opn5L1, but all-trans isomers of other retinoids are not

The Gi activation efficiencies of cell membrane fractions containing Opn5L1 whose N-terminus was replaced with that of *X. tropicalis* Opn5m (Opn5L1N) were monitored after incubation with various concentrations of all-*trans*-retinal (black open circles in panel a), all-*trans*-retinoic acid (red open squares in panel b), or all-*trans*-retinol (blue open triangles in panel c). Filled symbols indicate the results from mock-transfected HEK293T cells. Data points represent mean values ± s.d. (n=3). The results clearly showed that only the aldehyde form of all-*trans* retinoid acts as an agonist of Opn5L1.

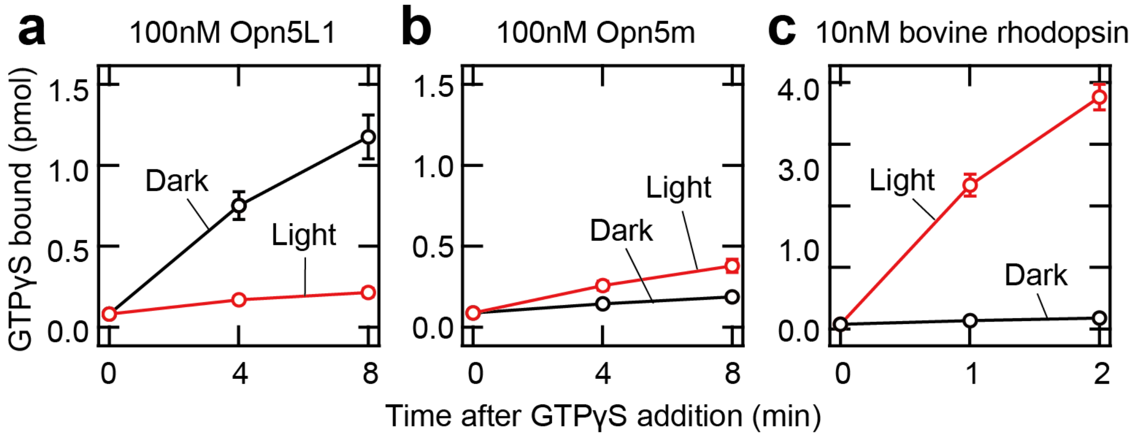

Supplementary Figure 5 Comparison of Gi activation efficiency of chicken Opn5L1 with those of bovine rhodopsin and chicken Opn5m

Gi activation efficiencies of chicken Opn5L1 (a), chicken Opn5m (b) and bovine rhodopsin (c) were measured in the dark (black line) and after >500 nm light irradiation for 1 min (red line) under identical conditions except for the concentration of opsin (100 nM for Opn5m and Opn5L1; 10 nM for rhodopsin) and incubation time (max. 8 minutes for Opn5m and Opn5L1; 2 minutes for rhodopsin). This assay was performed using DM-solubilized and purified samples at 0°C. The assay mixture contained 0.01 % DM, 1 μM [^{35}S]GTPγS, 140 mM NaCl, 8 mM $MgCl_2$, 1 mM DTT, 50 mM HEPES (pH 7.0), 4 μM GDP, 0.6 μM Gi, and opsin. It should be noted that the “Dark” sample contains 11-*cis*-retinal in Opn5m and rhodopsin; all-*trans*-retinal in Opn5L1. Based on light dependent changes of the slopes of these traces, the relative G protein activation efficiencies of Opn5L1, Opn5m and rhodopsin are estimated to be 1, 0.2, and 149, respectively. Data points represent mean values ± s.d. (n=3).

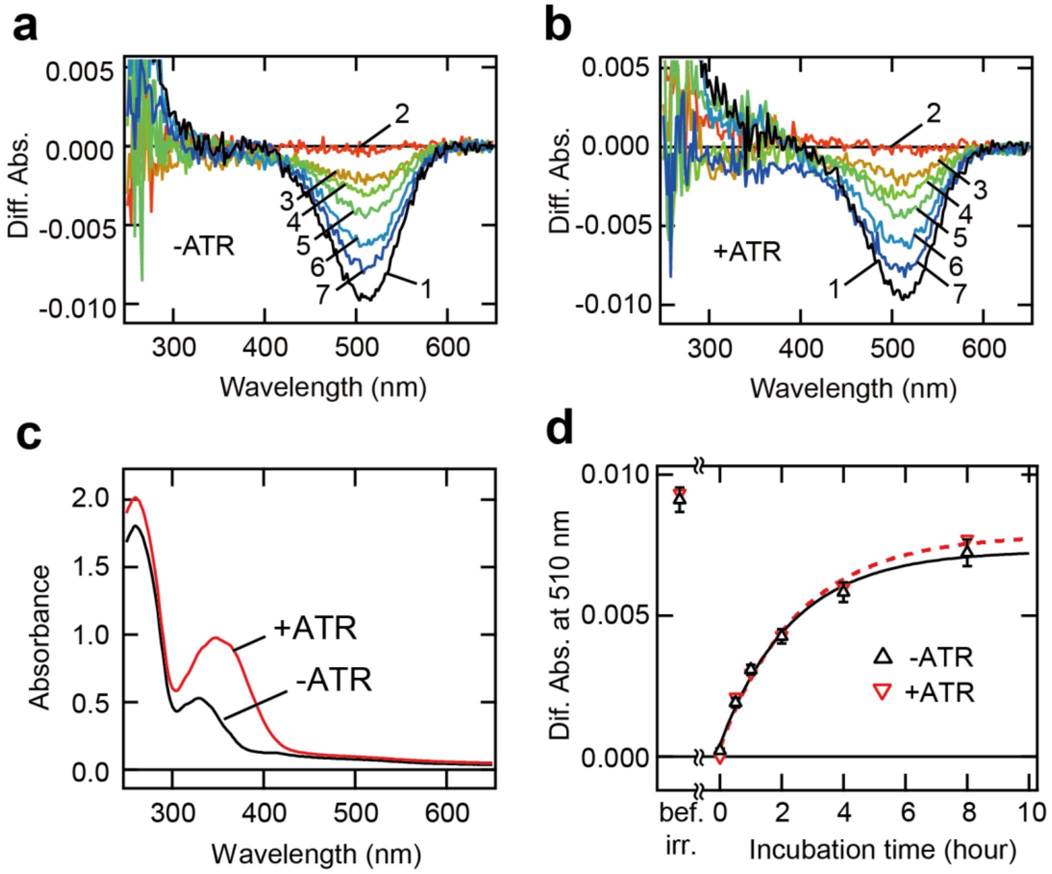

Supplementary Figure 6 Addition of all-*trans*-retinal did not affect recovery kinetics of Opn5L1

To assess the possibility of re-entry of exogenic retinal, additional all-*trans*-retinal was applied to a membrane suspension containing Opn5L1N bound to all-*trans*-retinal, and the mixture was incubated in the dark at 37°C after light irradiation. (a, b) Increase of photobleachable component having absorption maximum at 510 nm by incubation at 37 °C. First, membrane fraction containing Opn5L1N bound to all-*trans*-retinal was irradiated by >500 nm light for 2 min on ice. Irradiated samples were kept in the dark at 37 °C for the indicated times with or without additional all-*trans*-retinal at 40 μM (b and a, respectively). Then, Opn5L1N was extracted with the detergent dodecyl maltoside. Finally, the

recovered fraction was evaluated by measurement of the spectra before and after light irradiation in the presence of hydroxylamine at 20 mM. Hydroxylamine was added to avoid spectral perturbation by photoreaction of excess all-*trans*-retinal. The difference spectra between the spectra measured before and after >500 nm light irradiation for 2 min are shown. All the spectra were measured at 10 °C. Curve 1 was derived from the sample before on-ice light irradiation and incubation at 37 °C. This corresponds to the data point at bef. irr. in panel d. Curves 2-7 were derived from the samples extracted after 0, 0.5, 1, 2, 4, 8 hours incubations at 37 °C. (c) Example of absolute spectra of the extract of membrane fraction in the presence of hydroxylamine at 20mM. The spectrum of the sample with additional all-*trans*-retinal (+ATR) shows a significant increase of retinal oxime compared to the control (-ATR). (d) Kinetics of the recovery of the dark state of Opn5L1 with ("+ATR", reversed red triangle) and without additional all-*trans*-retinal ("-ATR", black triangle). Data points represent mean values ± s.d. (n=3). Single exponential functions fitted to the recovery data exhibited time constants of 2.57 (red broken curve) and 2.33 (black solid curve) hours, respectively. This shows that exogenous all-*trans*-retinal does not affect the kinetics of the recovery reaction of Opn5L1, that is, the reaction is not due to incorporation of retinal.

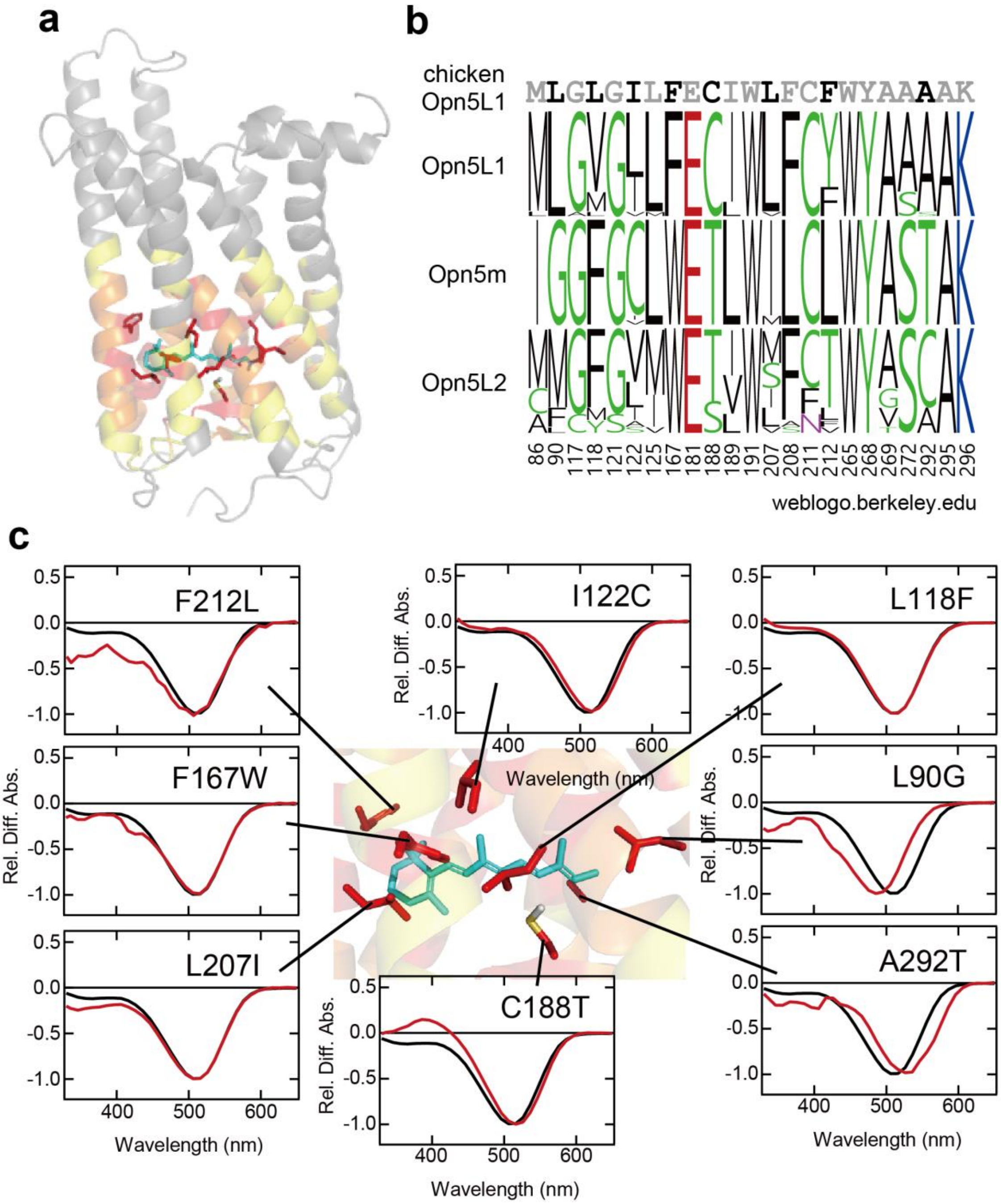

Supplementary Figure 7 Identification of the amino acid residue responsible for the unique photoreaction of Opn5L1

(a) Homology model of Opn5L1 that was constructed based on the X-ray crystallographic structure of bovine metarhodopsin II (PDB:3PXO) using Swiss-model server[3,4]. (b) A Weblogo sequence logo that was generated from

the amino acid residues within 4 Å from all-*trans*-retinal for each Opn5 sub-group[5]. Amino acid sequences of Opn5L1, Opn5m, and Opn5L2 groups were aligned by MAFFT. Eight amino acids were selected as well-conserved residues in the Opn5L1 subgroup (black letters in chicken Opn5L1 line). Residue numbers shown in the figure are based on the bovine rhodopsin numbering system. (c) The 8 amino acids of Opn5L1NC were replaced with the corresponding residues in chicken Opn5m, respectively. These mutants were expressed in HEK293T cells in the presence of all-*trans*-retinal, and difference spectra calculated from the spectra before and after irradiation were obtained. Red and black curves in each panel are the difference spectra obtained from the mutant and wild-type Opn5L1NC, respectively. It should be noted that the mutants were analyzed in crude extracts and that the difference spectrum of wild-type was obtained from a purified sample. Only the C188T mutant showed light-dependent formation of a product having a difference absorption maximum at about 390 nm, suggesting that the C188T mutation changed the photoreaction and absorption characteristics of the stable photoproduct of Opn5L1.

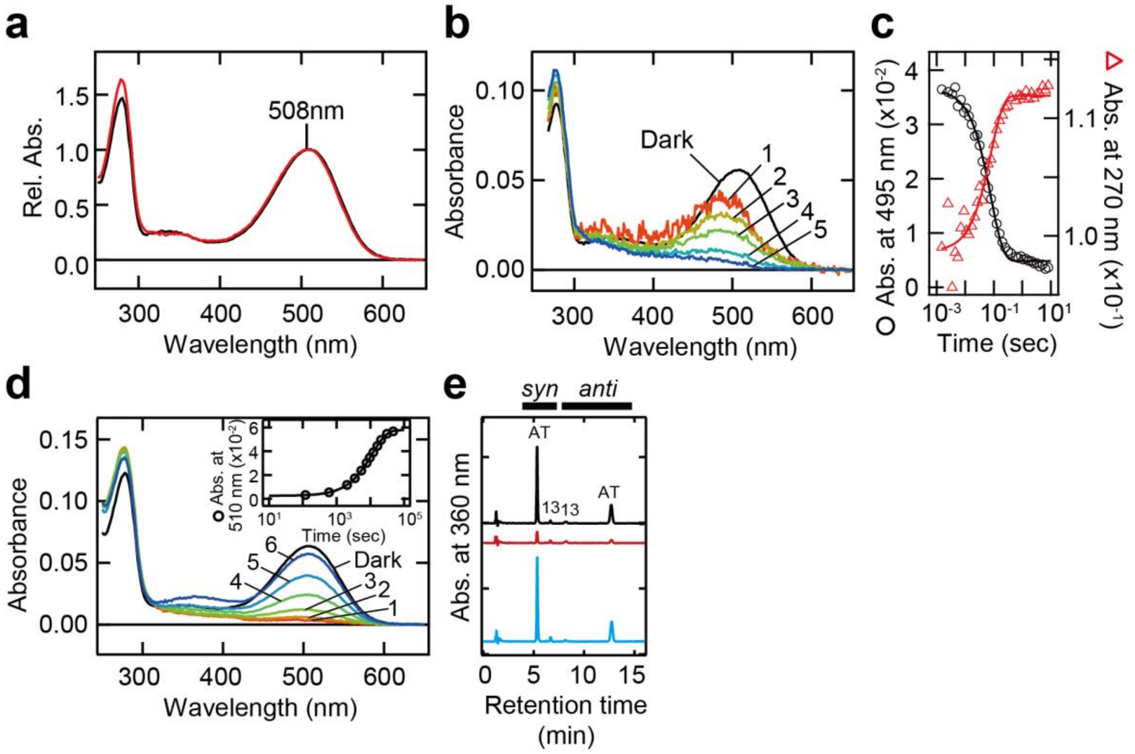

Supplementary Figure 8 Molecular characteristics of Opn5L1NC E177K/Q192K mutant

(a) Absorption spectra of Opn5L1NC E177K/Q192K (red) and WT (black) bound to all-*trans*-retinal. The absorption maximum of E177K/Q192K is slightly blue shifted to 508 nm compared to that of WT (λ_{max}=510 nm). (b) Absorption spectra of Opn5L1NC E177K/Q192K before (Dark) and 0.0015, 0.017, 0.05, 0.16, and 2.1 sec (curves 1-5, respectively) after >500 nm flash light irradiation at 37 °C. Unreacted component was subtracted from each measured spectrum after irradiation. (c) The absorbance at 495 nm (black circles) and 270 nm (red triangles) plotted against the time after flash irradiation. The time profiles were fitted by single exponential functions with the same time constant (solid curves, τ=0.081 sec). (d) Absorption spectra before (Dark) and 0, 8, 32, 88, 200, and 792 minutes (curves 1-6, respectively) after >500 nm light irradiation for 2 min at

37°C. (inset) The absorbance at 510 nm plotted against the time after irradiation (black circles). The data were fitted by a single exponential function (solid curve, τ=1.1 × 10^4 sec). (e) HPLC patterns of retinaloximes extracted before (black) and 2 min (red) or 13 hours (cyan) after light irradiation.

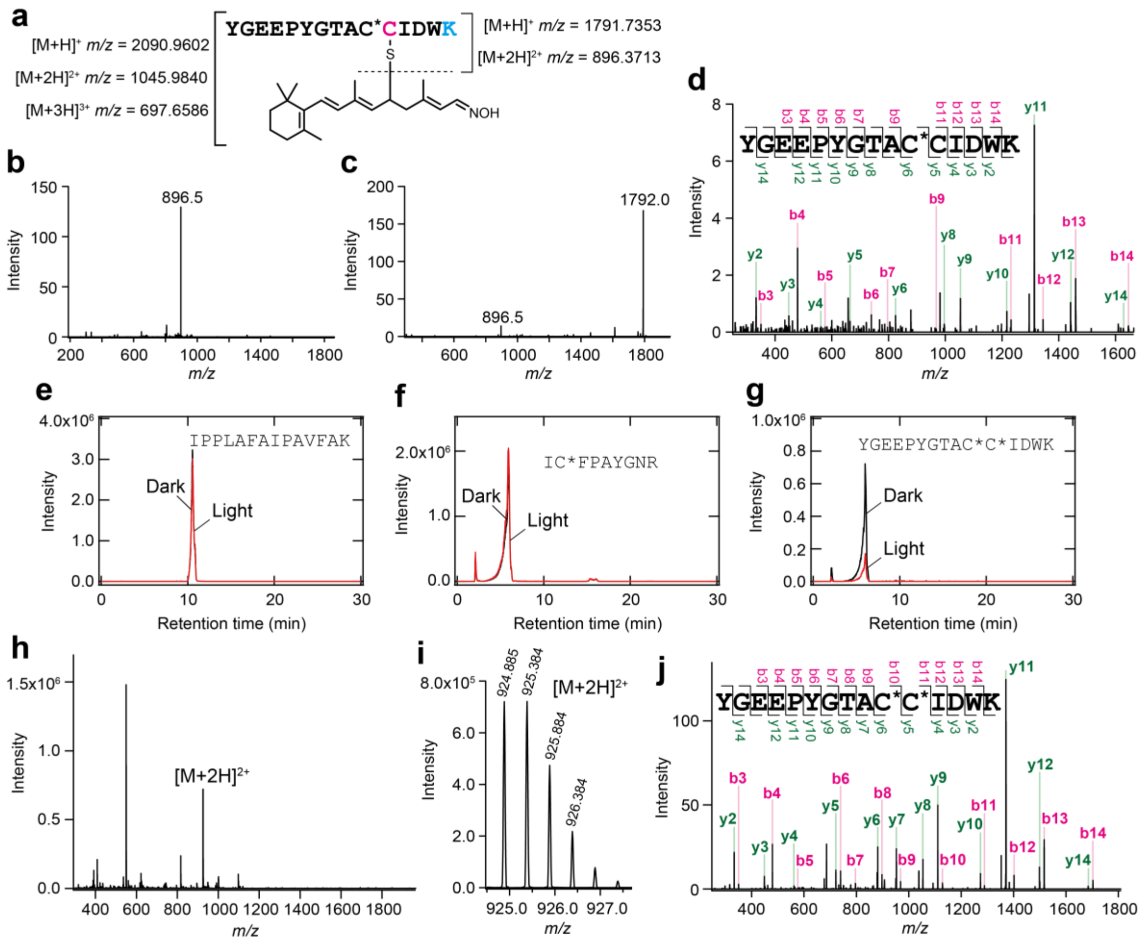

Supplementary Figure 9 Identification of the intermolecular adduct formation by LC-MS experiments

(a) Structure and possible ions of the expected peptide. Broken line shows the cleavage site of first-generation CID fragmentation. Asterisk indicates that the cysteine residue at this position was carbamidomethylated. (b, c) MS/MS spectra of the precursor ion at *m/z* 697.658 in Fig. 4d (b) and the precursor ion at *m/z* 1045.987 in Fig. 4e (c) in the main text, respectively. The spectra are averages of 22 independent spectra obtained from the precursor ions. Fragment ions observed at *m/z* 896.5 and 1792.0 are in good agreement with *m/z* values of $[M+2H]^{2+}$ and $[M+H]^{+}$ of YGEEPYGTAC*CIDWK, respectively, which are shown in a. This could be interpreted as the result of a predominant cleavage at

the carbon-sulfur bond between retinaloxime and cysteine by first generation CID fragmentation. (d) MS/MS/MS spectra of the precursor ion at *m/z* 896.5 in panel b. The spectra are averages of 22 spectra obtained from the precursor ion. Observed b- and y- series ions are annotated. (e, f) Examples of the peptides derived from tryptic cleavage at lysine and/or arginine residues. LC-MS profiles of *m/z* = 727.942 ± 0.05 (e) and 549.264 ± 0.05 (f) obtained from the light-irradiated (red) and the dark-adapted (black) samples are shown. These signals correspond to the isotopic *m/z* of the $[M+2H]^{+2}$ ions of the peptides IPPLAFAIPAVFAK (283-296) and IC*FPAYGNR (139-147), respectively. Respective ion signals were detected from the light-irradiated and the dark-adapted samples at nearly the same intensity. This shows that the efficiencies of the peptide preparation from the light-irradiated and the dark-adapted samples were nearly equal. (g) LC-MS profiles of *m/z* = 924.882 ± 0.05 corresponding to the isotopic *m/z* of $[M+2H]^{2+}$ of the peptide YGEEPYGTAC*C*IDWK. The traces obtained from the light-irradiated (red) and the dark-adapted (black) samples are shown. The intensity of the detected ion was decreased by light-irradiation. (h) Mass spectrum recorded at the retention time of 6.01 min, peak time of *m/z* = 924.882 ± 0.05. The mass signal of $[M+2H]^{2+}$ ion of YGEEPYGTAC*C*IDWK is specified. (i) Enlarged view of the mass signal of $[M+2H]^{2+}$ from panel h. (j) MS/MS spectra of the precursor ion at *m/z* 924.885 in panel i. Eighty-eight spectra obtained from the precursor ions were averaged. Observed b- and y- series ions are annotated.

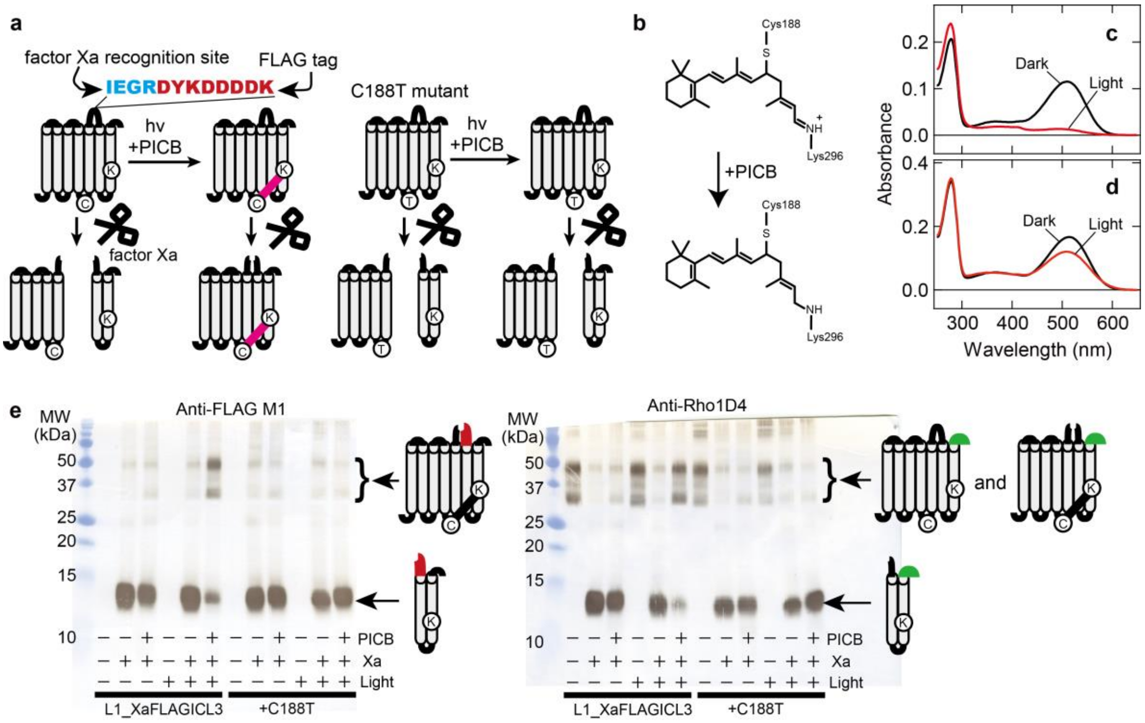

Supplementary Figure 10 Western blotting experiment to detect the intramolecular adduct

(a) Experimental design for detection of light-dependent intramolecular adduct by western blotting. To divide the Opn5L1 protein into two fragments and detect the cleavage at this position, we constructed Opn5L1NC containing a factor Xa recognition site and a FLAG tag sequence in intracellular loop 3 (Opn5L1_XaFLAGicl3). We also constructed its C188T mutant as a negative control. Purified pigments were cleaved into C-terminal and N-terminal fragments by factor Xa. The full-length protein is expected to be detected only when the pigment is irradiated in the presence of 2-picoline borane (PICB) for the formation of a stable retinal-thiol adduct connecting C188 and K296. Molecular weights of full-length and N-terminal fragment are expected to be 38016 and 11268 Da in the absence of any post-translational modifications. (b) Reaction scheme of the formation of retinyl-amine-thiol adduct by reducing

retinylidene-imine-thiol adduct with PICB. A reducing reagent, PICB, was used for amination of the imine linkage between retinal and K296[6]. Hydrolysis of the imine linkage between retinal and K296, and recovery to all-*trans*-retinal and cysteine from the retinal-thio adduct were blocked through this treatment. (c, d) Absorption spectra of Opn5L1_XaFLAGicl3 (c) and C188T (d) mutant. Black and red curves show the absorption spectra of the all-*trans*-retinal-bound state before and after light irradiation with >500 nm for 2 min, respectively. We confirmed that photochemical and subsequent thermal reactions of these proteins were similar to those of the original Opn5L1NC and its C188T mutant. All the spectra were recorded at 10 °C. (e) Detection of the intramolecular adduct by western blotting. Schematic views of visualized fragments are shown on the right-hand side. Epitopes are colored and thickened.
Opn5L1_XaFLAGicl3 was processed with factor Xa protease, and analyzed by western blotting by using anti-FLAG M1 monoclonal antibody and anti-rhodopsin Rho1D4 monoclonal antibody. Binding of anti-FLAG M1 antibody requires a free N-terminal FLAG epitope, and therefore only a fragment cleaved by factor Xa was visualized in this experiment[7]. On the other hand, Rho1D4 antibody can detect both the cleaved C-terminal fragment and the full-length protein that contains Rho1D4 epitope added at the C-terminus[8]. Accordingly, the wild-type sample processed by factor Xa after light irradiation in the presence of PICB showed a decrease of the amount of the C-terminal fragment band and an increase of the amount of the full-length protein bands, whereas the C-terminal fragment band was predominantly detected in other samples with factor Xa treatment. The full-length bands observed by using anti-FLAG M1 antibody

clearly show that the peptide bond between the factor Xa recognition site and the FLAG tag was successfully cleaved, but the N- and C-terminal fragments were connected through retinal-thiol adduct. Furthermore, an increase of the amount of the full-length protein bands was not observed in the C188T mutant in the corresponding lane. These results clearly demonstrate that light-stimulated Opn5L1 forms an intramolecular adduct connecting the N- and C-terminal fragments that are otherwise separated by cleaving intracellular loop 3.

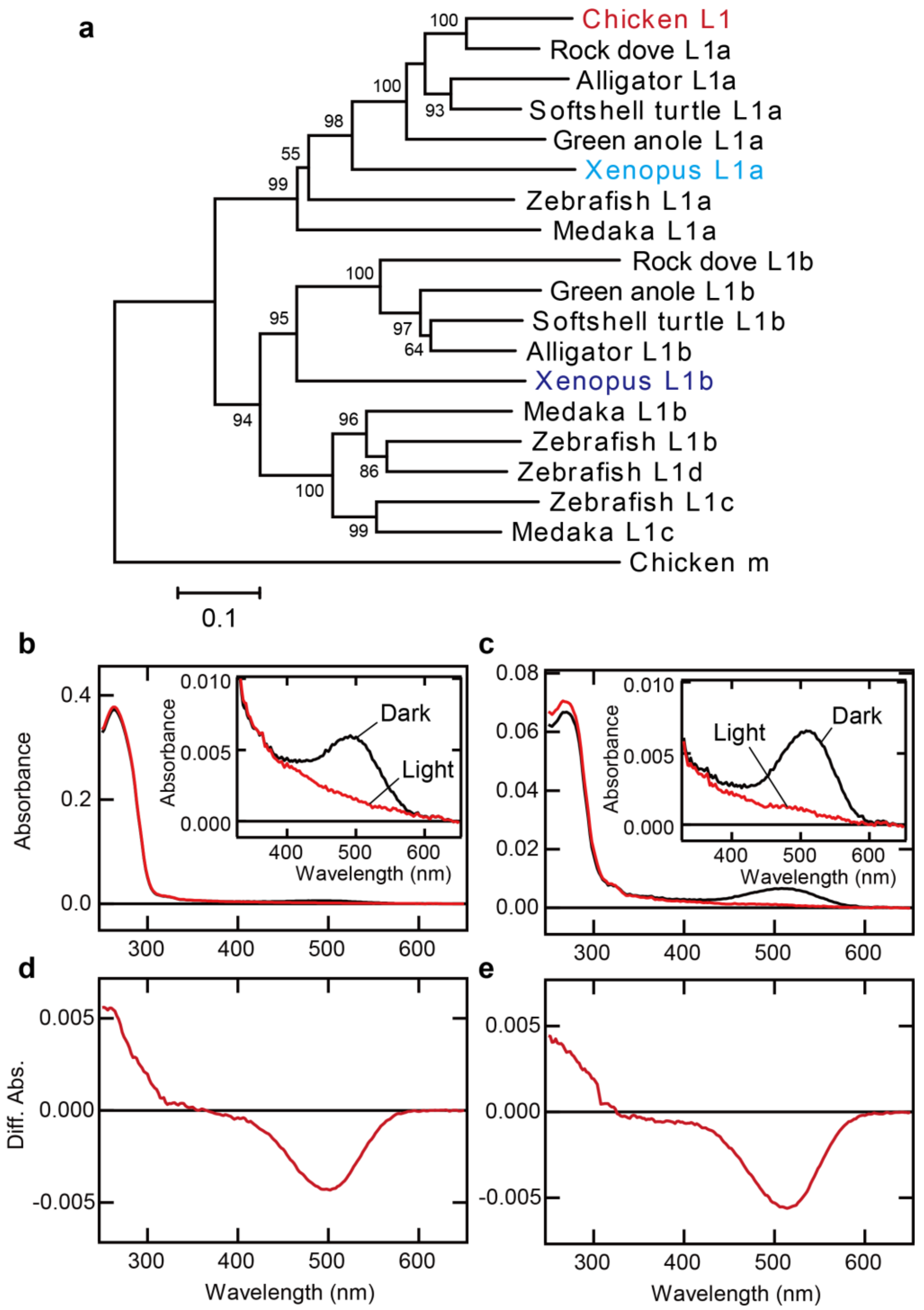

Supplementary Figure 11 Phylogenetic tree of Opn5L1 group and absorption spectra of Xenopus Opn5L1a and b

(a) The phylogenetic tree of the Opn5L1 subgroup. The tree was constructed based on the amino acid sequences using the neighbor-joining method. Chicken Opn5m was included as an outgroup. NCBI accession numbers are as follows:

chicken Opn5L1, NP_861418; rock dove Opn5L1a, XP_005504290; rock dove Opn5L1b, XP_005502781; alligator Opn5L1a, XP_006276218; alligator Opn5L1b, XP_006267486; softshell turtle Opn5L1a, XP_006113671; softshell turtle Opn5L1b, XP_006129533; green anole Opn5L1a, XP_003223576; green anole Opn5L1b, XP_003215407; Xenopus Opn5L1a, XP_002934176; Xenopus Opn5L1b, NP_001072846; zebrafish Opn5L1a, XP_698966; zebrafish Opn5L1b, NP_001038457; zebrafish Opn5L1c, XP_002666449; zebrafish Opn5L1d, XP_001340602; medaka Opn5L1a, XP_004079421; medaka Opn5L1b, XP_004071003; medaka Opn5L1c, XP_004070802 ; chicken Opn5m, NP_001124215. (b, c) Absorption spectra of Xenopus Opn5L1a (b) and Opn5L1b (c) reconstituted with all-*trans*-retinal, respectively. Spectra were recorded before (black curve) and after (red curve) irradiation with >500 nm light for 2 min. Insets are enlarged views in the visible region. (d, e) Difference spectra calculated from the spectra recorded before and after irradiation of Xenopus Opn5L1a (d) and Opn5L1b (e), respectively. Xenopus Opn5L1a and Opn5L1b were expressed in HEK293T cells similarly to chicken Opn5L1, and purified using dodecyl maltoside detergent. Light irradiation of these pigments caused disappearance of absorbance in the near-UV and visible wavelength region, and an increase in absorbance at wavelength near 270 nm, indicating that these pigments show photoreactions similar to that of chicken Opn5L1.

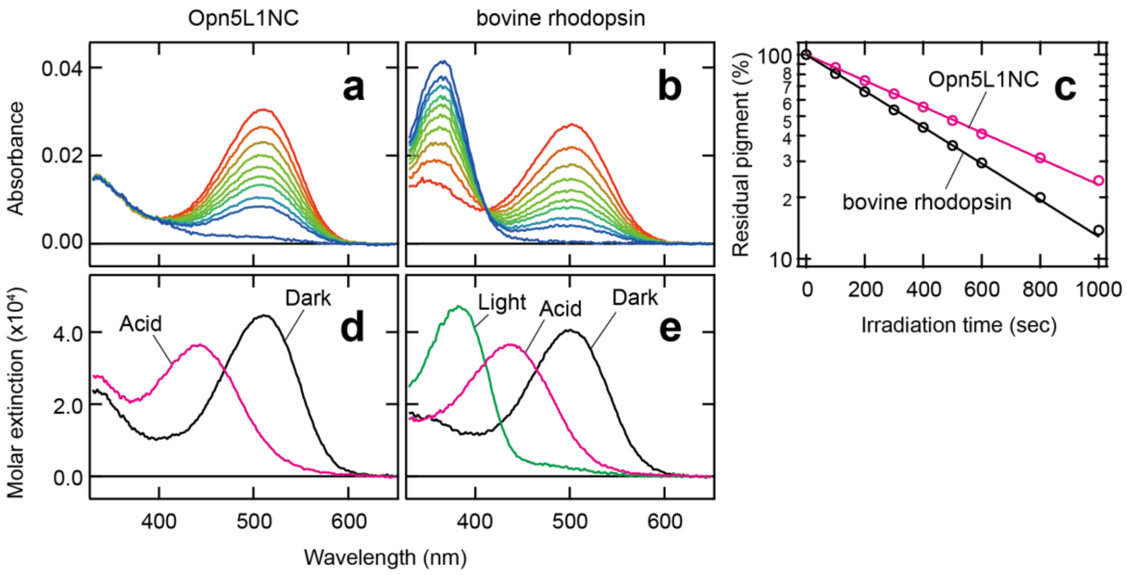

Supplementary Figure 12 Comparison of the photosensitivity and the molar extinction coefficient of Opn5L1 with those of bovine rhodopsin

(a, b) The absorption spectra of Opn5L1NC (a) and bovine rhodopsin (b) after successive irradiation with light passed through a bandpass interference (peak transmittance: 500 nm) and neutral-density filters. The absorption spectrum was measured before and after each irradiation at pH 7.0 and 0 °C. Finally, the samples were completely bleached by >500 nm light irradiation. The bovine rhodopsin sample was supplemented with hydroxylamine to remove any intermediates after irradiation. (c) The relative peak absorbance of Opn5L1NC (magenta open circles) and bovine rhodopsin (black open circles) were plotted against irradiation duration on a semi-logarithmic scale. Based on the linear regression of those plots, the photosensitivity of Opn5L1 was estimated to be 0.73 relative to that of bovine rhodopsin. (d, e) The absorption spectra of Opn5L1NC (d) and bovine rhodopisn (e) before and after acid denaturation. Because the dark state of Opn5L1NC contains all-*trans*-retinal as its chromophore, bovine rhodopsin was irradiated with >530 nm light for 6 min

before denaturation (magenta curve in panel e). Opn5L1NC and light-irradiated bovine rhodopsin were denatured by the addition of hydrochloric acid (green curves), which would result in the production of denatured pigment having protonated all-*trans*-retinal Schiff base. Thus, all the spectra were scaled so that the peak absorbance of denatured pigments from Opn5L1NC and bovine rhodopsin were the same. Based on the molar extinction coefficient of rhodopsin (40600 M^{-1} cm^{-1} at 500nm)[9], the molar extinction coefficient of Opn5L1 was estimated to be 44700 M^{-1} cm^{-1} at 510nm. Using the values of photosensitivity and molar extinction coefficient, the quantum yield of Opn5L1 was estimated to be 0.42 relative to that of bovine rhodopsin (0.65)[10]

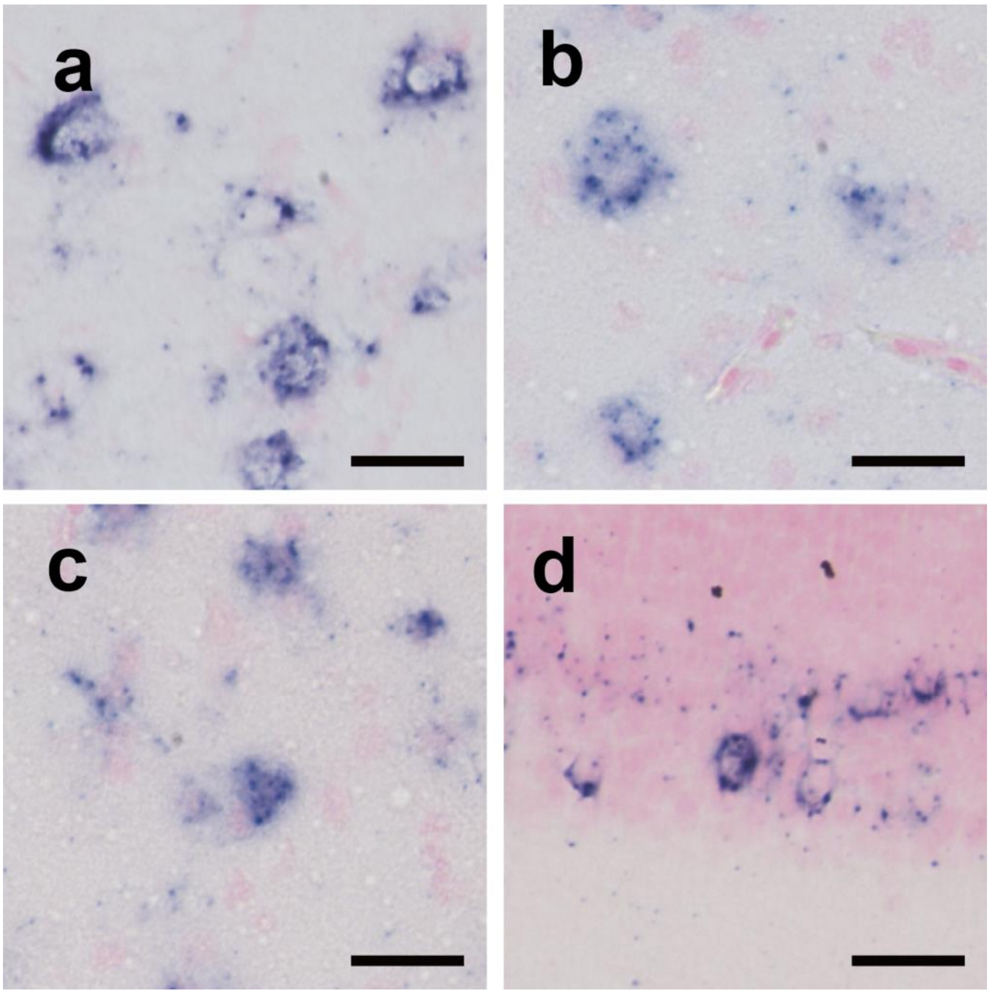

Supplementary Figure 13 High-power field microscopic images of *in situ* hybridization signals

The hybridization signals shown in Fig. 5b, d, f, and h in the main text were magnified and imaged by 60× objective lens (total 600× magnifications). Panels a, b, c, and d correspond to Fig.5b, d, f, and h, respectively. Scale bars: 20 μm.

Primer name	Sequence
Anchor primer	GGCCACGCGTCGACTAGTAC
cOpn5L1_5race	ACAGGCAAATGTGTTTCCCATACAA
cOpn5L1_3race1	TGGGATGCCACCTTCAAGAC
cOpn5L1_3race2	GAAGTGACACAGGGCAGTAG
Oligo-dT_3sites_primer	CTGATCTAGAGGTACCGGATCCTTTTTTTTTTTTTTTTTTTT
3sites_primer	CTGATCTAGAGGTACCGGATCC

Supplementary Table 1 Primer sequences for 5’- and 3’-RACE

5’RACE was carried out by using cDNA Amplification Kits for Rapid Amplification of cDNA Ends (Clontech) according to the manufacturer's instructions with the anchor primer provided in the kit and the gene specific primer cOpn5L1_5race. 3’RACE was carried out by two rounds of PCR amplification. The first PCR was performed on chicken brain whole cDNA (ZYAGEN) using cOpn5L1_3race1 and Oligo-dT_3sites_primer as primers. The second PCR was performed on the diluted first-round PCR product using cOpn5L1_3race2 and 3sites_primer as primers.

References

1. Katoh, K. & Standley, D. M. MAFFT multiple sequence alignment software version 7: improvements in performance and usability. *Mol. Biol. Evol.* **30,** 772–780 (2013).
2. Tamura, K., Stecher, G., Peterson, D., Filipski, A. & Kumar, S. MEGA6: Molecular Evolutionary Genetics Analysis Version 6.0. *Mol. Biol. Evol.* **30,** 2725–2729 (2013).
3. Choe, H.-W. *et al.* Crystal structure of metarhodopsin II. *Nature* **471,** 651–655 (2011).
4. Biasini, M. *et al.* SWISS-MODEL: modelling protein tertiary and quaternary structure using evolutionary information. *Nucleic Acids Res.* **42,** W252-258 (2014).
5. Crooks, G. E., Hon, G., Chandonia, J.-M. & Brenner, S. E. WebLogo: a sequence logo generator. *Genome Res.* **14,** 1188–1190 (2004).
6. Sato, S., Sakamoto, T., Miyazawa, E. & Kikugawa, Y. One-pot reductive amination of aldehydes and ketones with α-picoline-borane in methanol, in water, and in neat conditions. *Tetrahedron* **60,** 7899–7906 (2004).
7. Einhauer, A. & Jungbauer, A. Affinity of the monoclonal antibody M1 directed against the FLAG peptide. *J. Chromatogr. A* **921,** 25–30 (2001).
8. Molday, R. S. & MacKenzie, D. Monoclonal antibodies to rhodopsin: characterization, cross-reactivity, and application as structural probes. *Biochemistry* **22,** 653–660 (1983).
9. WALD, G. & BROWN, P. K. The molar extinction of rhodopsin. *J. Gen. Physiol.* **37,** 189–

200 (1953).

10. Kim, J. E., Tauber, M. J. & Mathies, R. A. Wavelength dependent cis-trans isomerization in vision. *Biochemistry* **40,** 13774–13778 (2001).
